# Spatiotemporal evolution and stage-driven mechanisms of population-land urbanization coupling coordination in counties of the yangtze river delta urban agglomeration: An empirical analysis based on multi-source remote sensing data

**DOI:** 10.1371/journal.pone.0356988

**Published:** 2026-08-26

**Authors:** Fei Tong, Yinxi Cai

**Affiliations:** 1 School of Financial Technology Applications, Zhejiang Financial College, Hangzhou, China; 2 School of Digital Economy and Trade, Wenzhou Polytechnic University, Wenzhou, China; Military University of Technology Faculty of Civil Engineering and Geodesy: Wojskowa Akademia Techniczna im Jaroslawa Dabrowskiego Wydzial Inzynierii Ladowej i Geodezji, POLAND

## Abstract

The coupled and coordinated development of population and land urbanization represents the core goal of new-type urbanization. However, existing research lacks refined measurement of the population-land urbanization coupling relationship at the county scale and insufficiently reveals the dynamic stage characteristics of its driving mechanism. To address this gap, this study takes counties in the Yangtze River Delta urban agglomeration as the research object. It integrates multi-source remote sensing data, including VIIRS NTL, LandScan population grid, CLCD land cover, and POI to construct a progressive analytical framework of fusion-extraction-assessment. First, the study uses wavelet transform to optimize the fusion of multi-source data. Second, it applies the U-Net deep learning model to finely extract the spatial patterns and temporal evolution characteristics of county land and population urbanization. Third, it uses a coupling coordination model to quantitatively evaluate the coupling matching relationship between the two from 2013 to 2025. Finally, it employs geographic detectors to identify the key driving factors and their explanatory power differences for coupling coordination development in different periods. The results show that the expansion speed of land urbanization in counties of the Yangtze River Delta urban agglomeration significantly exceeds that of population urbanization. Counties across the entire region exhibit varying degrees of population-land mismatch. The population-land urbanization coupling coordination degree of counties overall shows a steady upward trend. Spatially, it presents an evolutionary characteristic of spreading from point-like agglomeration in core cities to planar diffusion in the eastern region. The geographic detector results reveal a stage characteristic with dominant factors in different periods: foreign investment in 2013, economic development level in 2017, government expenditure in 2021, and industrial structure in 2025. This study meticulously reveals the spatiotemporal evolution patterns of population-land urbanization coupling coordination at the county scale. It identifies the dynamic evolution logic of population-land coordinated development in Yangtze River Delta counties. The study also constructs a technical framework for county urbanization research using multi-source remote sensing data fusion. This framework breaks through the limitations of single-indicator measurement and enhances the spatial precision and mechanism explanatory power of county urbanization research. This study provides empirical support for improving county-level spatial governance and promoting the coordinated development of population and land urbanization. It holds significant importance for enriching urbanization theory research and serving the implementation of the new-type urbanization strategy.

## 1. Introduction

With the acceleration of globalization, urbanization has become an important driving force for socio-economic transformation and spatial restructuring [1]. Urbanization generally includes two dimensions: population urbanization and land urbanization. However, research shows that globally, the pace of land urbanization is significantly faster than that of population urbanization, leading to a misalignment in the population-land relationship, a phenomenon particularly prominent in developing countries [2]. Therefore, in-depth exploration of the relationship between population and land urbanization not only helps reveal the coordination and inherent tensions in population-land interaction, but also provides an important scientific basis for optimizing territorial spatial governance and promoting new-type urbanization.

The essence of the coupling and coordinated development of population-land urbanization is the concrete practice of the theory of the geographical system of population-land relations in the field of urbanization. Its core lies in the matching and interaction of the two core elements of population and land in space, time and function. The county, as the basic unit of the population-land relationship areal system, serves as a key carrier for analyzing the population-land coupling coordination relationship [3]. The population-land misalignment and imbalanced spatial development at the county scale highly align with the theory of unbalanced regional development. The polarization and diffusion effects of core cities change over different stages, directly influencing the coordination level between population agglomeration and land development in counties. This constitutes a core cause of spatial differences in the county population-land relationship [4]. The coordinated development of population-land under the background of new urbanization is a practical requirement of the theory of urban-rural integration. It emphasizes promoting the two-way flow and spatial integration of urban and rural factors through the coordinated allocation of elements of population and land, and thereby solving the problem of population-land misplacement in counties [5]. These three theories together constitute the core theoretical framework for the research of county population-land urbanization coupling and coordination, which provides a theoretical basis for the research design, pattern analysis and mechanism explanation of this study.

The county, as a key spatial unit connecting national strategy and grassroots governance, is not only a main carrier for land expansion and population agglomeration, but also an important measure for regional coordinated development [6]. However, existing research at the macro level often focuses on the overall patterns of provinces or urban agglomerations, while generally neglecting the differentiated characteristics at the county level [7]. This makes the understanding of the relationship between county population and land urbanization still one-sided and limited, and the refined study of county-scale population-land coupling and coordination is still in the exploration stage.

Current research on urbanization mainly develops around three aspects. First, at the macro level, studies focus on urbanization rate, urban population proportion, and urban system evolution to reflect overall development levels and stage characteristics [8,9]. Second, at the spatial level, scholars examine urban spatial expansion, functional zoning, and urban-rural pattern transformation, thereby revealing the spatial restructuring during urbanization [10,11]. Third, at the quality level, research emphasizes the comprehensive impact of urbanization on economic growth, social governance, and ecological environment, highlighting the transition from speed-oriented to quality-oriented development [12–14]. Specifically, population urbanization primarily concerns the migration, relocation, and agglomeration of people between urban and rural areas; this process not only reflects changes in population size and density but also indicates the spatial reorganization of socio-economic behaviors such as employment, residence, and consumption [15,16]. In contrast, land urbanization is directly manifested as the expansion of construction land and the adjustment of functional structures, which can reveal the intensity of urban spatial development and land use efficiency [17]. Overall, these two aspects are interdependent and together constitute the fundamental concept of urbanization, yet they differ and create tension in terms of development speed and spatial manifestation [18].

In the study of population urbanization, early research primarily relies on macro indicators such as the urbanization rate, the proportion of urban permanent residents, and household registration migration to reflect the scale and speed of rural-urban population transfer, and emphasizes its role in economic growth and urban system evolution [19,20]. However, these measurement-methods based on statistical data have significant limitations in spatiotemporal resolution, making it difficult to reveal the spatial heterogeneity and dynamic evolution characteristics of population agglomeration [21]. With the development of spatial data acquisition and analysis technologies, the academic community begins to introduce geographic information systems, spatial econometric models, and remote sensing methods [22,23]. By analyzing population density patterns, agglomeration degrees, and migration flow models, it explores the spatial organization and evolution process of population urbanization [24,25]. Building on this foundation, research in recent years further deepens and begins to focus on the actual use behavior of population in urban space. This is reflected not only in the examination of population size and density, but also in the emphasis on analyzing the spatial orientation and functional demands of population in aspects such as production, residence, consumption, and travel [26–28]. Correspondingly, in terms of research data, the study of population urbanization has extended from traditional censuses and statistical yearbooks to geographical big data such as LandScan, POI and other population grids, realizing the transformation from static measurement of population size to dynamic portrayal of population behavior [29–33]. In summary, relevant empirical research has evolved from solely focusing on population expansion and density agglomeration to emphasizing the interaction between population activity spatial structure and functional zoning, thereby promoting a profound expansion of this field from static scale to dynamic behavior [34].

As for the study of population urbanization, as one of the most direct spatial manifestations in the urbanization process, traditionally, its research primarily relies on indicators such as construction land scale and proportion to reflect the expansion speed and intensity of urban space [35]. Specifically, early studies mostly use statistical yearbooks and land use change survey data to calculate construction land area and its growth rate, thereby revealing the expansion trend of land elements during urbanization [36]. However, such research is often limited to characterizing quantitative expansion and finds it difficult to deeply reveal deeper issues such as land spatial structure, functional differentiation, and utilization efficiency [37]. With the development of remote sensing monitoring and spatial analysis methods, land urbanization research gradually shifts towards exploring spatial patterns and structural characteristics [38]. Specifically, on one hand, the academic community widely uses multi-temporal remote sensing images and land cover data to quantitatively depict the spatiotemporal trajectory of construction land expansion through spatial analysis methods [39,40]; on the other hand, combined with NTL remote sensing data to pay attention to the functional transformation of land use, enabling multi-dimensional research that extends from quantitative expansion to functional intensity [41,42]. For example, research uses nighttime light remote sensing data to characterize development intensity and economic agglomeration levels, and employs methods such as geographically weighted regression to reveal their spatial heterogeneity and driving factors [43,44]. Overall, land urbanization research has developed from an early single-dimension focus into a multi-dimensional system centered on quantitative expansion, spatial patterns, and functional intensity [45,46].

Although the existing research has gradually paid attention to the coupling relationship on population-land urbanization, there are still many specific problems that have not been solved in the research on the county scale, and there are obvious shortcomings in the research methods: First, at the measurement level, it relies more on single statistical indicators such as urbanization rate and construction land area, which can only reflect the scale characteristics of population and land urbanization, it is difficult to refine the behavioral characteristics of population agglomeration and the functional strength of land development. Consequently, they fail to precisely measure the degree and spatial characteristics of population-land misalignment at the county scale. Second, at the scale level, research focuses more on the macro scales such as provinces and urban agglomerations, pays insufficient attention to the differentiated characteristics of counties, which are basic governance units. This creates an analytical bias of macro homogenization and fails to reveal the spatial heterogeneity and evolutionary differences in the population-land relationship among counties. Third, at the mechanical level, it focuses more on the static identification of driving factors, and lacks dynamic evolutionary analysis of driving factors at different stages of development, which fails to explain the stage-wise evolutionary logic of coordinated population-land development in the county level. Fourth, at the methodological level, some studies only use remote sensing data or statistical data alone, lack the integration and verification of multi-source data, and the combined application of coupling coordination model and spatial detection methods is insufficient, making it difficult to achieve the integration analysis of pattern portrayal – relationship evaluation – mechanism identification. In response to the above research gaps, this study uses multi-source remote sensing data and comprehensive spatial analysis methods to carry out systematic research from the county scale, and fills the existing research gaps through refined measurement, dynamic evaluation and institutional identification.

The combined application of multi-source remote sensing data, coupling coordination model and geographical detector is the best path to solve the research problem of county-scale population-land urbanization. Its suitability to the research needs of county-scale and its advantages over traditional methods are very significant: first, multi-source remote sensing data can break through the limitations of low spatial resolution and spatial heterogeneity of traditional statistical data. By integrating NTL data, land coverage, population grid and other data, it can simultaneously characterize the quantitative expansion and functional strength of land urbanization, the scale aggregation and behavioral characteristics of population urbanization, thereby realize the refinement and spatial measurement of county urbanization, and make up for the measurement defects of traditional single statistical indicators [47]. Second, compared with traditional correlation analysis and simple regression models, the coupling coordination model can not only reflect the correlation between population and land urbanization, but also quantitatively evaluate the degree of interaction matching and coordinated development level of the two, accurately reveal the degree and characteristics of population-land misalignment in the county, and realize the upgrade from relationship analysis to coordinated evaluation [48] Third, the geographical detector does not need linear assumptions, and can quantitatively identify the explanatory power of different factors on the degree of coupling coordination through variance decomposition. At the same time, it reveals the interaction between factors, perfectly adapts to the characteristics of significant spatial heterogeneity in county scale and no clear linear relationship between variables, and can realize the stage dynamic identification of driving factors, making up for the application limitations of the traditional regression model [49].With the continuous advancement of China’s new urbanization strategy, the research perspective is gradually shifting down from macro scales such as provinces and urban agglomerations to the key unit of counties. The reason for this is that county urbanization not only directly relates to the quality improvement of national urbanization as a whole, but also serves as a fundamental link in promoting urban-rural integrated development [50]. Specifically, current research mainly develops along two dimensions: on one hand, the academic community examines changes in county population size, migration patterns, and employment structure adjustments from a population perspective, thereby revealing the interactive relationship between population agglomeration and urban development [51]; on the other hand, from a land perspective, it focuses on the expansion of county construction land, industrial land layout, and ecological space protection, deeply exploring issues of land use efficiency and sustainable development [52]. Additionally, some studies attempt to use spatial econometric models, system dynamics, and comprehensive evaluation indicator systems to quantitatively measure county urbanization levels and analyze their driving mechanisms and spatial differentiation patterns [53,54]. However, population urbanization and land urbanization do not always advance synchronously at the county level. In reality, some counties experience imbalanced situations of having land but no people or having people but no land, meaning that the speed of land development and expansion exceeds population agglomeration, or that population influx is rapid while land space carrying capacity is insufficient [55,56]. This population-land mismatch phenomenon not only restricts the quality improvement of county urbanization but also further intensifies urban-rural disparities and regional development imbalances [26].

In recent years, the academic community has gradually shifted from singular population or land perspectives to comprehensive research on their relationship, with an emphasis on revealing the coupling and coordination mechanisms between the two to systematically evaluate the rationality and sustainability of county urbanization [57]. This important shift not only deepens the understanding of the inherent patterns of county urbanization, but also provides new theoretical foundations and practical support for optimizing county spatial governance and improving relevant policy formulation.

Based on the above research background and research gaps, this study conducts empirical analysis with the county area of the Yangtze River Delta urban agglomeration as the research object. The core research goal is to integrate multi-source remote sensing and geographical big data, to meticulously characterize the spatiotemporal pattern of county population-land urbanization from 2013 to 2025, to quantitatively assess the coupling coordination characteristics and spatial differences between the two, to identify the key driving factors of coupling coordination at different developmental stages, and to reveal the dynamic evolutionary logic of coordinated population-land urbanization development in Yangtze River Delta counties. Thus, provide empirical support and policy reference for improving the spatial governance of the county and promoting the high-quality development of new urbanization. Focusing on the research objectives, this study puts forward three core research questions: the first is what are the characteristics of the spatiotemporal pattern of county population-land urbanization in the Yangtze River Delta urban agglomeration from 2013 to 2025, and whether there are spatial heterogeneity and stage changes in the population-land misalignment of the county scale? The second is how the coupling and coordination level of population-land urbanization in the Yangtze River Delta urban agglomeration has evolved, and what are its spatial distribution characteristics and difference laws? The third is what are the key driving factors that affect the coupling and coordination of county population-land urbanization in the Yangtze River Delta at different stages of development, and what is the evolutionary logic and internal mechanism of its dominant role? The methodological innovation points of this study are reflected in two aspects: first, it constructs a county-level urbanization measurement framework of wavelet transformation data fusion and U-net deep learning, which breaks through the limitations of traditional single-indicator measurement, and realizes the spatial refinement extraction and portrayal of population-land urbanization; second, it develops a dynamic mechanism analysis method of coupling coordination model and geographical detector enabling the quantitative evaluation of the level of population-land coupling and coordination in the county and the accurate identification of driving factors at different stages, thus improves the analysis system of the coordinated development of urbanization in the county.

## 2. Materials and methods

### 2.1. Study Area

**Fig 1:**
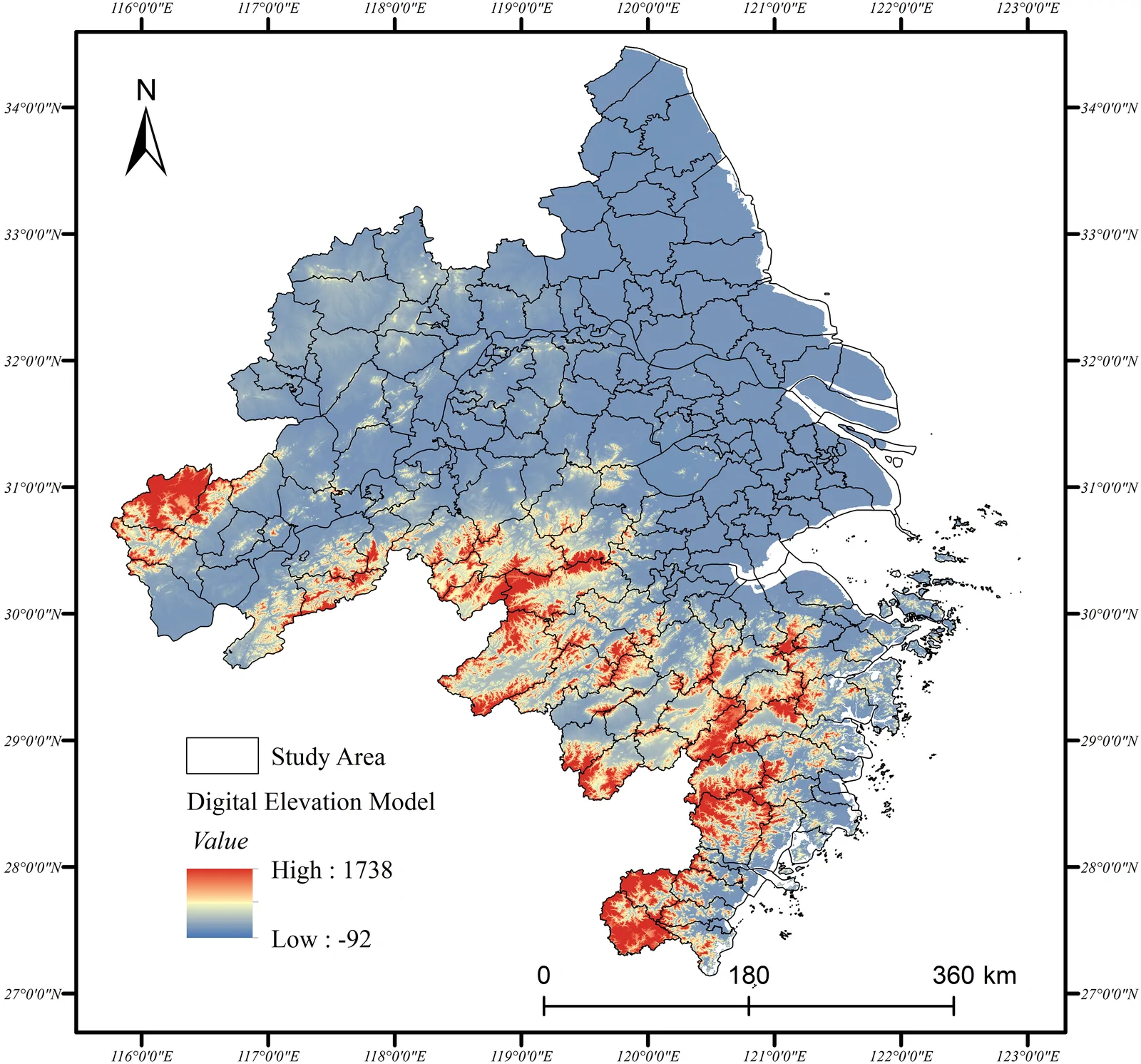
The Yangtze River Delta Urban Agglomeration (including a total of 41 prefecture-level cities and 192 county-level research units in Shanghai, Jiangsu, Zhejiang and Anhui). (Standard Map Service System of the Ministry of Natural Resources of China – Standard Map of China – Approval No. GS (2022) 1873).

The Yangtze River Delta urban agglomeration is one of China’s most economically dynamic and highly urbanized regions, with its counties playing a key role in supporting population agglomeration and land development (Fig 1) [58]. In recent years, although the overall urbanization of population and land in the region has progressed rapidly, it has shown a certain imbalance on the scale of the county: the population of some counties gathers rapidly and the land urbanization is relatively lagging behind, resulting in increased pressure on housing and infrastructure; while in other counties, there is too fast land expansion and insufficient population introduction phenomenon, resulting in the problems of empty cities and inefficient use of land. This population-land mismatch pattern not only affects the quality and efficiency of regional urbanization, but also poses new challenges to the coordinated development of urban and rural areas and spatial governance. Therefore, it is of great theoretical value and practical significance to reveal the coupling and coordination characteristics and driving mechanism of population urbanization and land urbanization in the Yangtze River Delta urban agglomeration from the county level.

This study selects four years of 2013, 2017, 2021 and 2022 to carry out spatiotemporal pattern analysis. The year selection is not set randomly, but takes into account the major national policy nodes, the characteristics of regional development stages, data availability and research design needs to jointly determine. 2013 serves as the starting baseline year. This year is an important turning point in China’s urbanization from rapid expansion to focusing on quality improvement. 2017 corresponds to a critical period for the in-depth implementation of the National New Urbanization Plan and the accelerated advancement of integrated development in the Yangtze River Delta urban agglomeration. This year can effectively reflect the evolutionary characteristics of the population-land relationship during the mid-term stage of urbanization. 2021 is both the first year of the 14th Five-Year Plan and a key year when the COVID-19 pandemic exerts a periodic impact on regional urbanization development. 2025 represents the most recent period.

In summary, the selection of the above years takes into account the national policy cycle, urbanization evolution stage, external shock events and data integrity, which can systematically reflect the stage evolution and long-term trend of the coupling and coordination of county population-land urbanization in the Yangtze River Delta from 2013 to 2025, and meet the needs of research design and spatiotemporal analysis.

### 2.2. Study data

The data used in this study include nighttime light (NTL) data, LandScan data, CLCD data, and POI data. NTL data represent the level of economic development, LandScan data represent population size, CLCD data reflect land use status, and POI data indicate how populations utilize space. We fuse NTL data with CLCD data to interpret land urbanization outcomes; this combination captures both the quantitative expansion of land development and the functional intensity of land use, thereby more accurately characterizing the spatial pattern and evolution of land urbanization. Similarly, we combine LandScan data and POI data to explain population urbanization outcomes, as their integration reflects population behavior in using urban space and agglomeration characteristics. These two approaches complement each other: they not only depict population size and distribution but also reveal the spatial structure and functional linkages of population activities, offering a more comprehensive representation of the essential nature of population urbanization. This study follows the processing process of unified extraction, standardized cleaning and multi-dimensional verification for all remote sensing data. The quality and applicability of the data are verified through quantitative indicators to ensure the reliability of the subsequent analysis results (see the subsection for the specific processing methods and verification results of each data).

#### 2.2.1. *NTL data.*

**Fig 2:**
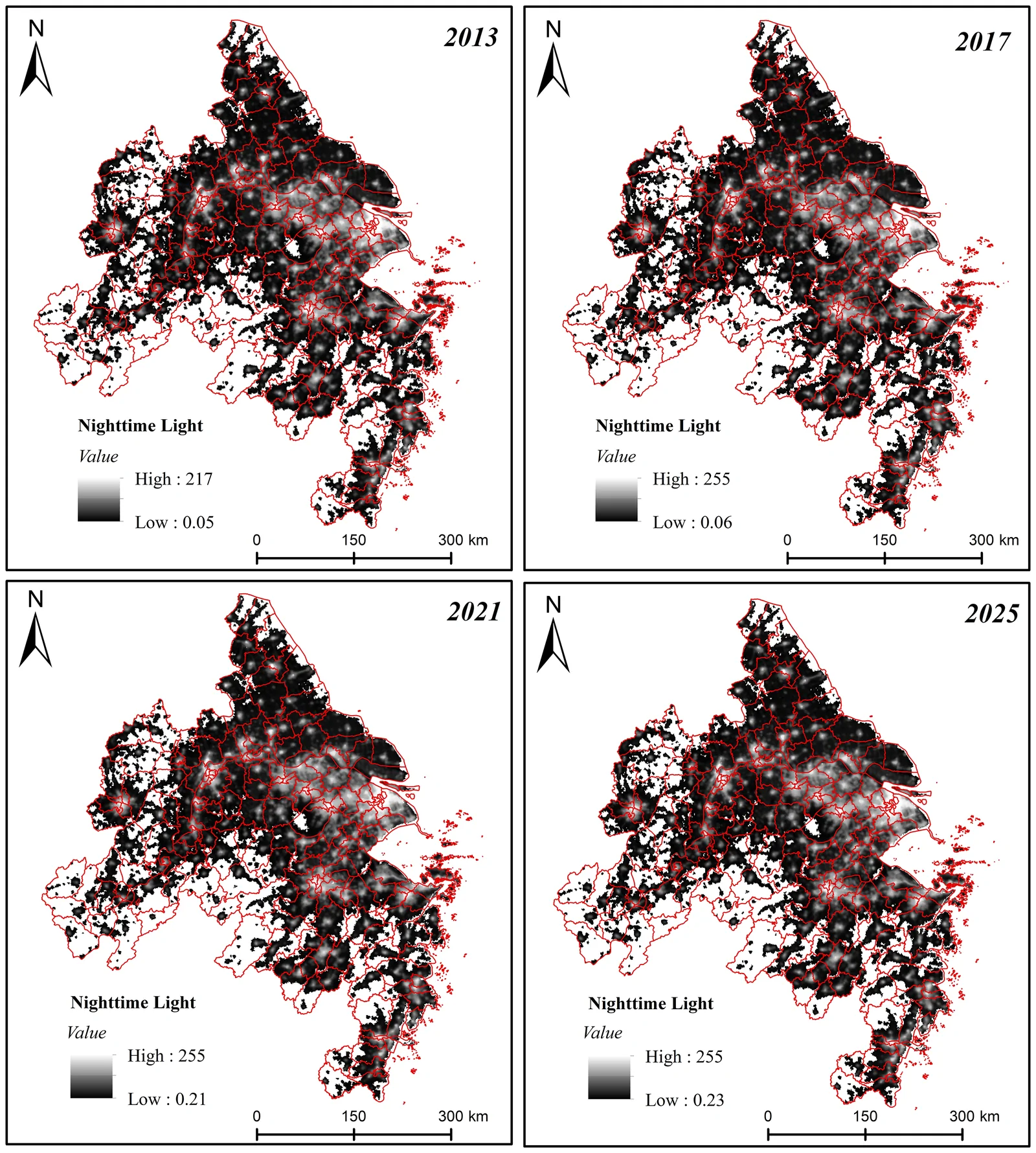
NTL Data across Counties in the Yangtze River Delta Urban Agglomeration from 2013 to 2025. (Standard Map Service System of the Ministry of Natural Resources of China – Standard Map of China – Approval No. GS (2022) 1873).

The primary dataset employed in this study comprises annual nighttime light (NTL) data at the county level within the Yangtze River Delta urban agglomeration, covering the period from 2013 to 2025. Sourced from the Visible Infrared Imaging Radiometer Suite (VIIRS) nighttime light products, these data feature a spatial resolution of 500 meters, with a time span from 2013 to 2025 and offer significant advantages including strong temporal continuity, extensive spatial coverage, and good integration compatibility with statistical datasets [59]. It can effectively characterize the level of regional economic development and the functional intensity of land development, which is one of the core remote sensing data to portray land urbanization. As complete annual data for 2025 are not yet publicly available, this study approximates the 2025 NTL intensity by averaging the full-year 2024 data with available data from January to August 2025. The processing process of specific data is as follows. First of all, based on the vector boundary of the county administrative division of the Yangtze River Delta city agglomeration, ArcGIS 10.8 is used to extract the original grid data of VIIRS, and the NTL data within the research area is cropped. The unified projection coordinate system is uniformly set to WGS_1984_UTM_Zon. e_50N to ensure the consistency of spatial consistency. Secondly, in order to eliminate the impact of sensor noise, cloud pollution and abnormal image elements on the results, the quantile method and neighborhood analysis method are used for data cleaning: 1. The 0.5% extreme image element (brightness value >80 nW·cm − ^2^·sr − ¹) is eliminated by the quantile method to avoid isolated high-value point interference. 2. We use a 3 × 3 neighborhood window to interpolate and fill anomalous low-value pixels (brightness value = 0) to ensure data continuity. 3. The multi-phase image is spliced and inlaid to eliminate the image stitching, and finally obtains the NTL grid data sequence of the continuous timing from 2013 to 2025 [60]. Finally, the correlation analysis with statistical data is used for verification, and the annual GDP data of each county in the Yangtze River Delta urban agglomeration is selected as a reference to calculate the Person correlation coefficient between the average brightness value of night lighting and GDP. The results show that R^2^ = 0.89 (P < 0.01), indicating that the NTL data can effectively characterize the level of regional economic development, and the data quality meets the research requirements. The resulting spatial distribution of NTL intensities across counties in the Yangtze River Delta urban agglomeration from 2013 to 2025 is visually presented in Fig 2.

#### 2.2.2. *Landscan Data.*

**Fig 3:**
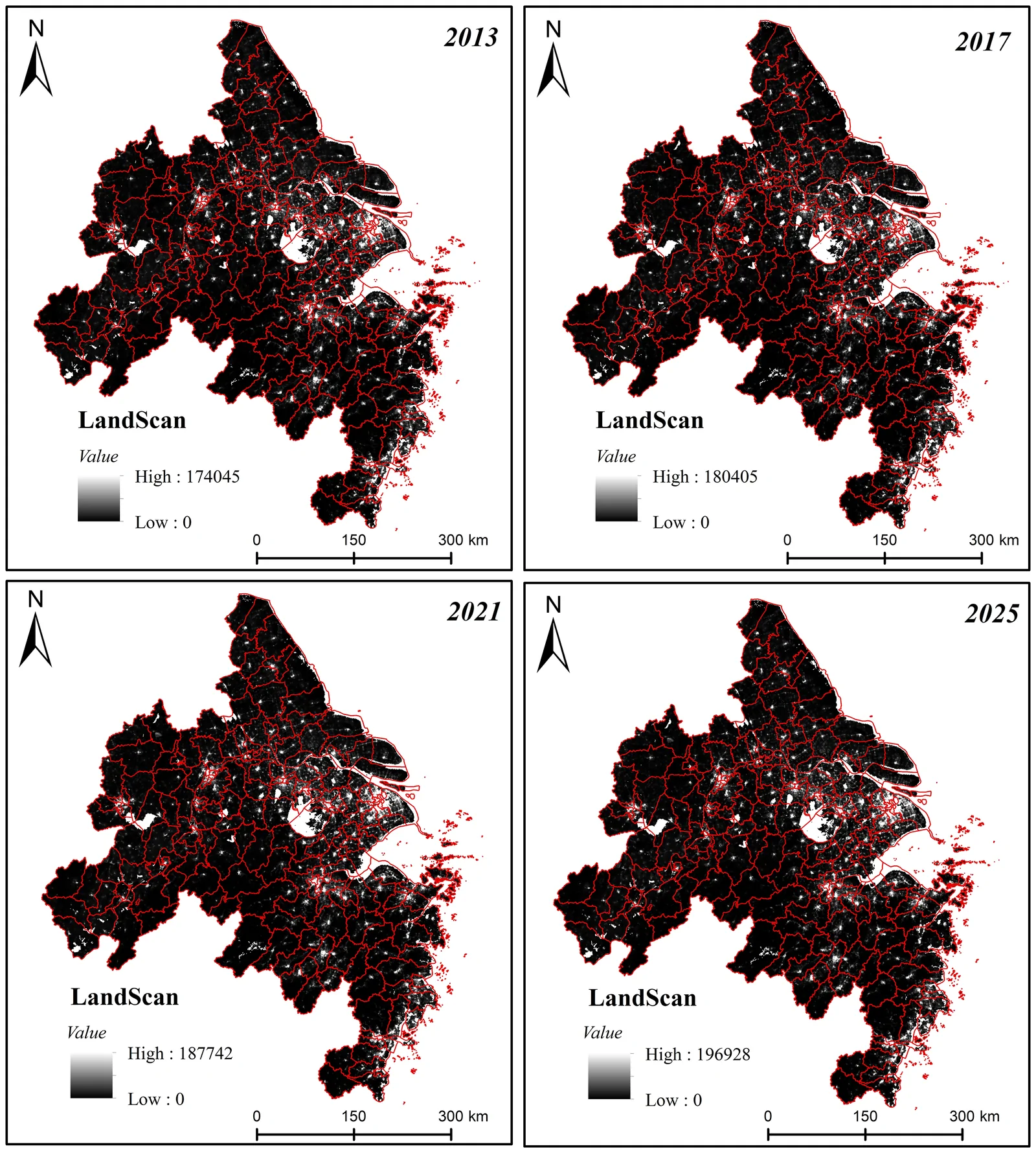
LandScan Data across Counties in the Yangtze River Delta Urban Agglomeration from 2013 to 2025. (Standard Map Service System of the Ministry of Natural Resources of China – Standard Map of China – Approval No. GS (2022) 1873).

The second part of the data used in this study comprises the LandScan global population distribution dataset for the period 2013–2025. For consistency, the 2025 data are similarly constructed as the average of full-year 2024 values and available January–August 2025 data. Developed and released by the Oak Ridge National Laboratory (ORNL) in the United States. The time span of the data is from 2013 to 2025, with a spatial resolution of 1 km. The data integrates multi-source information such as census, road traffic, land use, remote sensing images, etc., and combines with the probability distribution model to realize the refined defiction of population distribution [61]. Unlike conventional population statistics, LandScan integrates multi-source information—including census data, road networks, land use, and remote sensing imagery—with a probabilistic allocation model to generate spatially refined population estimates. This approach offers distinct advantages in terms of dynamic updating, broad geographic coverage, and cross-regional comparability. The specific processing process of the data is as follows: First, based on the vector boundaries of the county administrative division of the Yangtze River Delta urban agglomeration, ArcGIS 10.8 is used to extract the original grid data of LandScan, crop the scope data of the research area, and unify the projection coordinate system as WGS_1984 _UTM_Zone_50N to maintain space matching with other data sources. Secondly, spatial resampling and abnormal value elimination are used for data cleaning: 1. We use bilinear interpolation to resample the data to a resolution of 500 m, which is consistent with the resolution of NTL data, which is convenient for subsequent fusion and analysis. 2. Eliminate abnormal image elements with population density value > 20,000 people / km^2^, such image element is mainly an isolated high value caused by data error, which has no practical geographical significance. 3. The boundary smoothing of the image element at the county boundary is carried out to avoid the sudden change in population density caused by administrative division. Finally, it is compared and verified with the census data, and the county resident population data of the seventh national census in 2020 is selected as a reference, and the relative error between the total population of LandScan data and the census data is calculated. The results show that the average relative error is 6.2%, and Kappa coefficient = 0.85 (P < 0.01), indicating that the data can accurately characterize the population distribution characteristics of the county and meet the research requirements. This study ensures the consistency of data with other data sources on the spatiotemporal scale through the above preprocessing [62]. This processed dataset provides a robust basis for examining population agglomeration patterns and urbanization processes at the county level within the Yangtze River Delta urban agglomeration. The resulting population distribution across counties from 2013 to 2025 is presented in Fig 3.

#### 2.2.3. *CLCD Data.*

The third component of the data employed in this study is the China Land Cover Dataset (CLCD), developed by Wuhan University, which provides annual land use and land cover information from 2013 to 2025 at a spatial resolution of 30 meters. The data covers 8 types of land use, including construction land, arable land, forest land, water bodies, etc., and the classification mode adapts to China’s regional geographical characteristics [63]. As the 2025 data are not yet available, the 2024 dataset is utilized as its substitute. Derived from the integration of multi-source remote sensing imagery, the CLCD offers detailed classification of various land cover types—including construction land, cropland, forest, and water bodies. This dataset not only features extensive geographical coverage and continuous temporal consistency, but its classification system is also particularly tailored to the Chinese context, enhancing its applicability for regional studies [64]. The specific processing process of the data is as follows: first, based on the vector boundary of the county administrative division of the Yangtze River Delta urban agglomeration, we use ENVI 5.3 and ArcGIS 10.8 for collaborative processing. We initially perform band combination and mask clipping on the original CLCD data to extract the data within the study area. Then, through a reclassification operation, we merge the eight land use types into two categories: construction land and non-construction land, highlighting the core research object of land urbanization. Secondly, we use precision optimization and grid aggregation for data cleaning: 1. We eliminate isolated patches with an area of <0.01 km^2^ in the construction land, which are pseudo-construction land caused by classification errors. 2. We use a 3 × 3 neighborhood windows to smooth the boundaries of the construction land to eliminate classification noise. 3. We aggregate the 30 m resolution construction land data to a 500 m resolution to match the resolution of the NTL data for subsequent fusion analysis, with the aggregation rule being a pixel is classified as construction land if the proportion of construction land within it exceeds 50%. Finally, the dual verification method of field verification and comparison with land use change survey data is adopted: 1. We select 100 typical county-level sample points in the Yangtze River Delta urban agglomeration for field survey and verification, achieving an overall accuracy of 92.3% for construction land. 2. A comparison with the construction land area data from the county-level annual land use change surveys yields a Pearson correlation coefficient of R^2^ = 0.94 (P < 0.01), indicating that the data accurately represents the characteristics of county-level construction land expansion and meets the research requirements.

**Fig 4:**
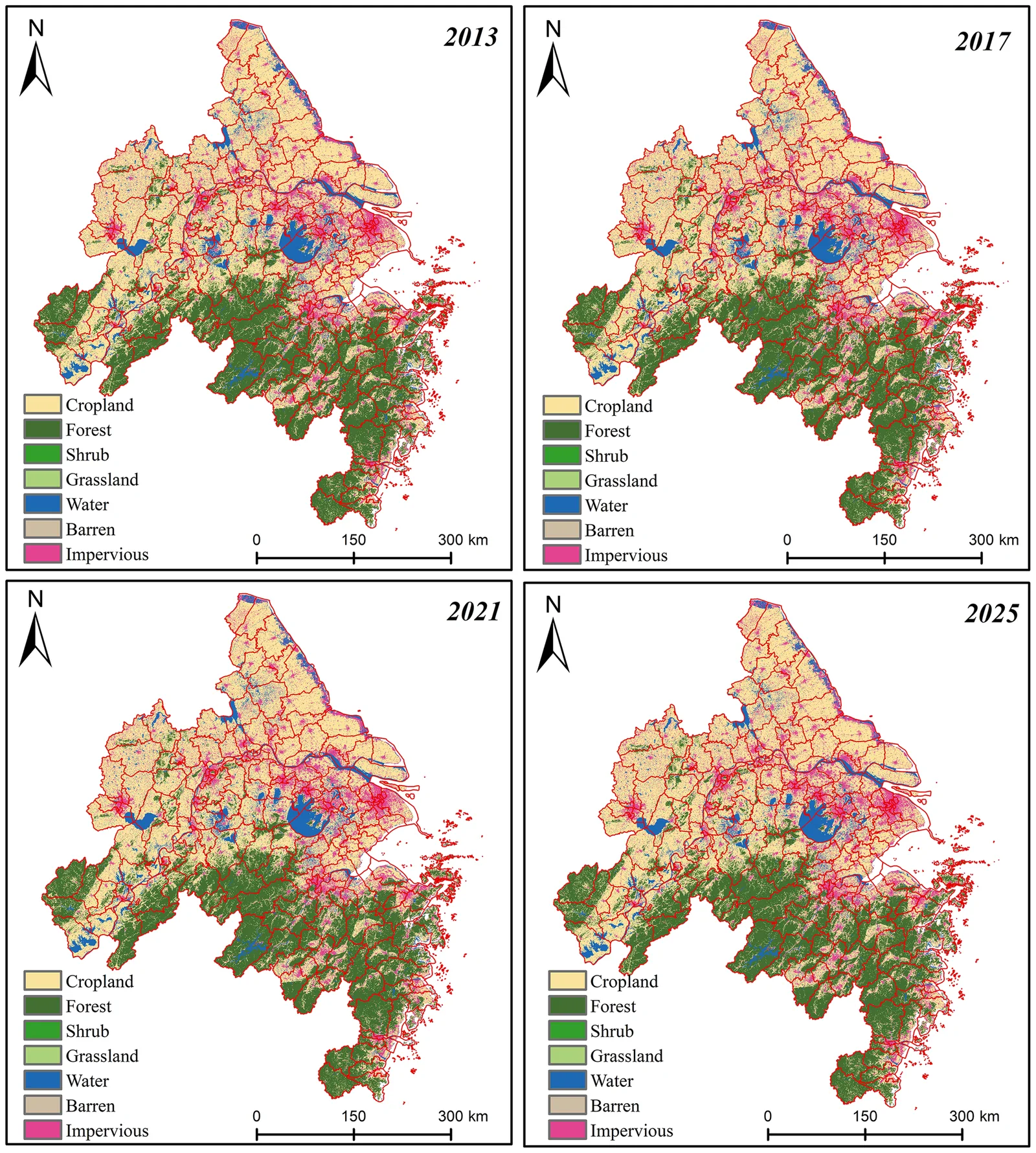
Land use classification across Counties in the Yangtze River Delta Urban Agglomeration from 2013 to 2025. (Standard Map Service System of the Ministry of Natural Resources of China – Standard Map of China – Approval No. GS (2022) 1873).

Through the above pretreatment, this study highlights the expansion and structural changes of construction land, which provides a reliable basis for the portrayal of the level of land urbanization in the county. The results of the land use classification for the counties in the Yangtze River Delta urban agglomeration are shown in Fig 4.

#### 2.2.4. *POI Data.*

The fourth dataset employed in this study comprises Points of Interest (POI) data for the Yangtze River Delta urban agglomeration spanning from 2013 to 2025, with the spatial accuracy for point features reaching 10 m for geographic coordinates. The data records the spatial location and functional types of residents’ production and living facilities, covering 12 categories and 86 sub-categories such as commerce, public services and transportation facilities [65], which can effectively reveal the spatial distribution and functional needs of population activities, and is a key data that characterizes the behavioral dimension of population urbanization. The POI data for 2025 is constructed from the average value of the data for the whole year of 2024 and the data from January to August 2025. The following is the specific data processing process. First of all, based on the vector boundaries of the county administrative division of the Yangtze River Delta urban agglomeration, the Python geospatial analysis library (GeoPandas) is used to extract the original POI data spatially to screen out the POI points within the scope of the research area. At the same time, according to the research needs, six categories of POI (commerce, residence, transportation, education, medical care, leisure) closely related to population activities are extracted, and administrative office, industry and other categories unrelated to the daily use of the population are excluded, focusing on the actual use of urban space by the population. Secondly, multi-step cleaning of de-weighting, coordinate calibration and abnormal value elimination is adopted: 1. remove duplicate POI points through the double matching method of names and coordinates, with an average de-weighting rate of about 15.3%. 2. Geographically calibrate the POI points of coordinate offset and match the electronic map of Gaode map to ensure the accuracy of spatial positioning. 3. Eliminate abnormal POI points with county overflow and missing coordinates, and finally obtain a standardized timing POI data set. 4. Use ArcGIS 10.8 to convert POI point data into grid density data with 500 m resolution, and calculate the number of POI in each grid to be consistent with the LandScan data resolution, which is convenient for subsequent integration and analysis. Finally, the correlation analysis with population density data is used for verification, and the county-scale POI density and LandScan population density are selected for analysis. The results show that the Person correlation coefficient R^2^ = 0.87 (P < 0.01), indicating that POI density can effectively characterize the intensity of population activity, which is highly consistent with the population distribution characteristics, and the data quality meets the research requirements.

**Fig 5:**
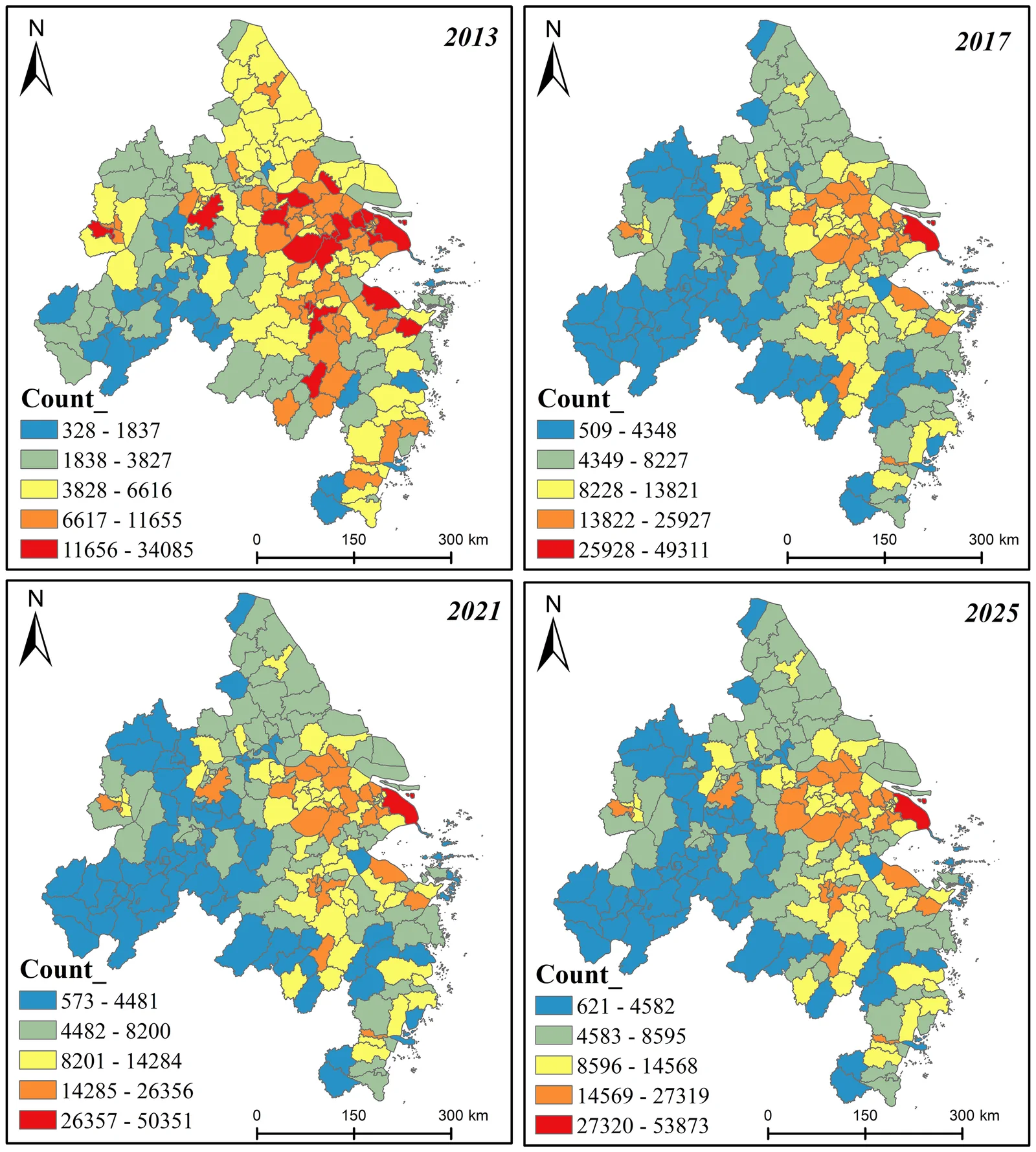
Spatial Distribution of POI data across Counties in the Yangtze River Delta Urban Agglomeration from 2013 to 2025. (Standard Map Service System of the Ministry of Natural Resources of China – Standard Map of China – Approval No. GS (2022) 1873).

This data provides important support for portraying the urbanization level of the county population from the functional and behavioral dimensions [66]. The spatial distribution of the final county POI data of the Yangtze River Delta from 2013 to 2025 is shown in Fig 5.

It should be noted that the NTL data and LandScan data used in this study are constructed from the average data of the whole year 2024 and the data from January to August 2025. The selection of this estimation method is based on the fact that the 2025 study period has not yet concluded, and high-resolution monthly remote sensing data is only updated up to August. Moreover, the urbanization development of the Yangtze River Delta urban agglomeration has a strong annual continuity. From 2013 to 2024, the inter-annual fluctuation coefficient of population-land urbanization is consistently less than 0.15. Therefore, using the average of the data from the recent 16 months can best approximate the actual development level for the full year of 2025. However, this method also has potential biases, namely, it does not cover the urbanization dynamics from September to December 2025. If unexpected situations, such as major land development or the implementation of population agglomeration policies, occur in counties during the fourth quarter, it may lead to minor deviations between the estimated data and the actual data. Moreover, such deviations may be slightly higher in counties with faster urbanization development compared to those with stable development.

The original spatial resolution of the four types of core data in this study is significantly different (CLCD land cover data has an original resolution of 30m, VIIRS NTL data has an original resolution of 500m, LandScan population data has an original resolution of 1000m, and POI data is scale-free point data). Combined with the analysis needs of the county scale, data characteristics and subsequent integration and modeling requirements, we follow the core principles of aggregating high-resolution to appropriate resolution, resampling low-resolution to appropriate resolution, and converting point data into a raster grid. Consequently, all data are uniformly standardized to a spatial resolution of 500m × 500m. The CLCD construction land raster data with a 30m resolution is subjected to raster aggregation processing, with the aggregation factor set to 17 (30m × 17 ≈ 510m, approximately matching the 500m resolution and serving as the optimal integer factor for raster aggregation in ArcGIS). The LandScan population density raster data with a 1000m resolution is resampled using the bilinear interpolation method, reducing the resolution from 1000m to 500m. Bilinear interpolation calculates the new pixel values through a weighted average of the four neighboring pixels, making it suitable for continuous population density data. This method effectively ensures the spatial continuity and numerical smoothness of the resampled data while avoiding the jagged raster boundary issues caused by the nearest neighbor method. For the POI point data, the process involves converting the scale-free point data into a raster grid.

Although the VIIRS NTL data, LandScan population grid, CLCD land coverage and POI geographical big data integrated in this study can portray the characteristics of population-land urbanization from multiple dimensions, there are still certain limitations due to data acquisition, technical processing and objective attributes: First, NTL data is susceptible to clouds, sensor noise and urban light pollution. Although it has been preprocessed by depolarization, noise elimination, etc., it still cannot completely eliminate the deviation of local image elements. Second, LandScan population data is the grid data simulated by the model, although the verification accuracy of the census data is relatively high, there is still a slight deviation from the actual resident population of the county, and it cannot accurately reflect the household registration attributes and flow frequency of the population. Third, POI data is limited by the scope of data collection and update frequency of commercial platforms, and there are omissions in the collection of facility points in remote areas of the county, and in the process of data de-weighting and classification integration, some niche function POI is eliminated, and it is difficult to fully reflect the full characteristics of population activities. But in general compared to relying on individual data sources, the integrated use of multi-source datasets effectively addresses the limitations of statistical data—such as their limited timeliness—and the narrow interpretability of single-type remote sensing data. This comprehensive approach provides more complete, objective, and reliable data support for systematically analyzing the coupling coordination characteristics and driving mechanisms between population and land urbanization at the county level within the Yangtze River Delta urban agglomeration.

### 2.3. Methods

This study constructs a progressive analytical framework that integrates multi-source data fusion, deep learning feature extraction, coupling coordination relationship assessment, and finally, mechanism identification using geographic detectors. Each method is interlinked and mutually supportive, with the output of preceding methods providing the core input data and analytical foundation for subsequent methods. The specific process correlations are as follows: First of all, complete the integration of multi-source remote sensing and geographical big data through wavelet transformation, solve the limitations of single data characterization characteristics, and generate land urbanization (NTL and CLCD) and population urbanization (LandScan and POI) integrated grid data. Secondly, the integrated data is entered into the U-Net deep learning model to complete the refined extraction of the spatial pattern and quantitative value of county-scale land urbanization and population urbanization to obtain the population urbanization index (U1) and land urbanization index (U2) of each county, which provides a quantitative basis for subsequent coupling and coordination analysis. Third, the urbanization index extracted from the U-Net model is substituted into the coupling coordination model, and the coupling degree (C) and coupling coordination degree (D) of the counties of the Yangtze River Delta urban agglomeration from 2013 to 2025 are calculated as dependent variables for geographical detector analysis. Finally, the driving factors of economic, social, spatial and other dimensions are selected as independent variables, and the coupling coordination and driving factors are jointly substituted into the geographical detector model to identify the key driving factors and differences in explanatory power of coupling and coordinated development in different periods. The following sections elaborate on the specific application of each method.

#### 2.3.1. *Data Fusion.*

Wavelet transformation is a multi-scale signal analysis method, which can be deployed in both time and frequency domains at the same time to decompose and reconstruct signals or images, so it is widely used in remote sensing image fusion. In this study, the wavelet transform is used to integrate two sets of core data: NTL data and CLCD data, LandScan population data and POI data, so as to give full play to the respective advantages of different types of data and improve the accuracy and reliability of land urbanization and population urbanization characterization. This study combines the characteristics of remote sensing grid data with the scale requirements of county urbanization research, determines the specific practical parameters and fusion rules of wavelet transformation, and completes the fusion quality verification through quantitative indicators. Its basic idea is to decompose images into small wavelet spaces of different scales, and realize the balance of details and overall characteristics through the complementary integration of high-frequency and low-frequency information. Compared with traditional fusion methods such as principal component analysis (PCA) and product transformation, wavelet transformation has the advantages of maintaining good spectral characteristics, fully expressing spatial details, and adapting to the characteristics of different data sources, which can effectively avoid spectral distortion and spatial resolution reduction in the fusion process [67,68]. The fundamental formula of wavelet transform is expressed as follows:


$$\begin{array}{c} f(t)=\sum_{j}\sum_{k}c_{j,k}\varphi_{j,k}(t) \end{array}$$

Here, f(t) represents the original image, while $\varphi_{j,k}(t)$ denotes the scaling and shifting of the wavelet basis function at scale j and position k, with $c_{j,k}$ being the corresponding wavelet coefficients. Through scale decomposition, the low-frequency components primarily capture the overall contour and trend of the image, whereas the high-frequency components contain edge and fine detail information. In data fusion, the low-frequency part is taken from the independent data to maintain the consistency of the main structure and characteristics; the high-frequency part is taken from the auxiliary data to enhance the spatial details and edge information, and finally the fused image is reconstructed through inverse wavelet transformation.

Considering the spatial resolution of the raster data (500m) and the analytical requirements at the county scale, this study determined through multiple experimental validations that the db4 wavelet basis function serves as the core basis function. This base function has the characteristics of good tight support, high smoothness and small reconstruction error, and adapts to the integration needs of multi-source remote sensing and geographical big data. At the same time, it sets up a three-layer wavelet decomposition not only ensures sufficient decomposition scale to extract the detailed characteristics of the data, but also avoids data redundancy and computational efficiency reduction caused by too many decomposition layers. The threshold value selection of the decomposition and reconstruction process adopts the soft threshold value method, and the threshold coefficient is set to 0.05 to suppress noise interference in the fusion process and to improve the smoothness of the fusion data. The study follows the fusion rules of master data to determine the trend and auxiliary data to supplement the fineness. After scale decomposition, the low-frequency coefficient of the master data and the high-frequency coefficient of the auxiliary data are reconstructed. Specifically: 1. The low-frequency coefficient retains the core characteristics of the master data, does not replace the coefficient, and only normalizes the alignment. 2. The high-frequency coefficient extracts the detailed information of the auxiliary data, weights it according to the preset weight, and then combines it with the low-frequency coefficient of the master data. 3. Finally, the reconstruction of the fusion image is completed through inverse wavelet transformation, generating fusion raster data with the overall characteristics of the master data and the detailed information of the auxiliary data, and the bilinear interpolation method is used to ensure the spatial continuity of the image element during the reconstruction process.

Based on the integration characteristics of the two groups of data, this study formulates differentiated whole-process operation specifications, as follows: in order to eliminate the differences in the outline of different data and ensure the effectiveness of the fusion, the extremely differential normalization processing of all raster data participating in the fusion is carried out, and the data value is uniformly mapped to the range from 0 to 1. The calculation formula is:


$$\begin{array}{c} Xnorm=Xmax-XminX-Xmin \end{array}$$

Where, Xnorm is the normalized data value, X is the original value of the data, and Xmax and Xmin are the maximum and minimum values of the data within the research area respectively.

Based on the research objectives, the division of primary and auxiliary data and the weight allocation for wavelet decomposition layers are determined. The low-frequency component, which reflects the core trends of the data, is assigned a higher weight, while the high-frequency component, representing detailed features, is assigned a lower weight. For each dataset, the sum of the weights for the low-frequency and high-frequency components is 1. The specific weight settings are determined by considering the detailed characteristics of land urbanization and population urbanization.

To ensure spatiotemporal consistency in subsequent analyses, all fused data are uniformly standardized to a 500m spatial resolution using bilinear interpolation for resampling. Raster aggregation followed a pixel-by-pixel calculation rule, where pixel values reconstructed through wavelet transform are integrated cell by cell. This process ultimately generates continuous raster datasets, serving as the foundational analytical data for land urbanization and population urbanization, respectively.

Finally, the fused image is obtained through inverse wavelet transformation reconstruction. This study verifies the spectrum and spatial quality of the integrated data. The results show that the average gradient of land urbanization fusion data increases by 42.6%, and the information entropy increases by 38.3%; the spatial detail recognition of population urbanization integration data increases by 45.2%, all of which show that the wavelet transformation fusion effectively preserves the core characteristics of each data source, enhancing the accuracy and reliability of urbanization characterization.

#### 2.3.2. *Deep Learning.*

U-net represents a classical fully convolutional neural network architecture, initially developed for medical image segmentation. Its key strength lies in performing precise pixel-level spatial feature extraction and classification. Unlike conventional convolutional neural networks, U-net employs a symmetric encoder-decoder framework supplemented with skip connections between corresponding layers. This design allows the model to comprehensively capture global semantic features during down-sampling, while progressively restoring spatial resolution through up-sampling and replenishing localized details via skip connections. Consequently, U-net effectively balances spatial localization accuracy with semantic representation capacity [69]. These characteristics make U-net particularly suitable for remote sensing applications such as land cover classification, change detection, and feature extraction.

The U-net architecture comprises four key components: (1) Encoder: consisting of multiple convolutional and pooling layers that progressively extract hierarchical features from input images, capturing macro-level semantic information relevant to urbanization processes; (2) Bottleneck layer: positioned at the network’s core, it employs deeper convolutional operations to model global contextual features; (3) Decoder: utilizing up-sampling and convolutional operations to gradually restore spatial resolution while reconstructing detailed information; (4) Skip connections: these directly transfer feature maps from corresponding encoder layers to the decoder, preserving edge details and spatial structures that might otherwise be lost during down-sampling. For model training, this study employs processed multi-source remote sensing data as input, with land urbanization and population urbanization classification results serving as supervised labels, establishing an end-to-end deep learning framework [70,71]. The detailed training procedure follows these steps:

Data Preparation and Partition: We construct separate labeled datasets for land urbanization and population urbanization. The sample data are partitioned into training, validation, and test sets following a 7:2:1 ratio to ensure model stability and generalization capability. Data Augmentation: Prior to training, we apply random augmentation techniques—including cropping, rotation, mirroring, and scaling—to the input images to enhance sample diversity and mitigate overfitting. Model Configuration: The model employs cross-entropy loss as the optimization objective and utilizes the Adam optimizer for iterative training. The initial learning rate is set to 1e-4 and dynamically adjusted via a cosine annealing schedule to balance convergence speed and training stability. All convolutional kernels are set to 3 × 3, with ReLU as the activation function and Softmax at the output layer for multi-class probability prediction. Training Procedure: The model is trained in a GPU environment with a batch size of 16. After each round of iteration is completed, evaluation indicators such as accuracy, intersection ratio (IoU) and Kappa coefficient are calculated on the verification set. Prevent over-fitting through the early stop strategy, that is, terminate the training when the accuracy of the verification set is no longer improved for 10 consecutive rounds. Model verification results: The overall accuracy of the land urbanization extraction model is 91.5%, IoU of 0.86, and the Kappa coefficient of 0.89; the overall accuracy of the population urbanization extraction model is 90.2%, IoU of 0.84, and the Kappa coefficient of 0.87, all of which achieve high extraction accuracy, indicating that the model can effectively realize the pixel-level extraction of the spatial pattern of county urbanization. In this study, the fused raster data is divided into training, validation, and test sets in a 7:2:1 ratio. Data augmentation is introduced to enhance the model’s generalization capability. The training epochs are set to 100, and the learning rate to 0.001. The model is trained using the cross-entropy loss function and the Adam optimizer, and training is stopped when the validation set loss does not decrease for 10 consecutive epochs to avoid overfitting. In order to verify the effectiveness of model training and the accuracy of the extraction results, this study selects the three core indicators of loss curve, overall accuracy (OA) and Kappa coefficient to verify the accuracy of the model output results of the test set. The meaning and verification results of each indicator are as follows: the loss value of the loss curve training set is reduced to 0. 082, the loss value of the verification set is reduced to 0.095, and the difference between the two is less than 0.02, indicating that there is no obvious over-mitting or under-mitting of the model, and the training effect is good. The overall accuracy of the test set of land urbanization extraction results reaches 92.36%, and the overall accuracy of the test set of population urbanization extraction results reaches 90.87%, both of which are higher than the high-precision threshold of 90%. The Kappa coefficient of the results of land urbanization extraction reaches 0.891, and the Kappa coefficient of the results of population urbanization extraction reaches 0.865, both of which are higher than the excellent level of 0.85, indicating that the results of the model extraction are highly consistent with the real urbanization pattern.

Model output and result explanation: After the training is completed, the U-net model can make pixel-level predictions on the input multi-source images and output the spatial distribution pattern of land urbanization and population urbanization.

The only and core application of the output results of the U-Net model in this study is the basic input data for coupling coordination index calculation, not independent verification results or simple auxiliary classification results. The output results of the model are divided into two categories: spatial distribution grid diagram and county quantitative index. Among them, the spatial distribution grid diagram is used to visually display the spatial pattern characteristics of county population-land urbanization and provide visual support for the spatial and spatial pattern analysis of research results. The county quantitative index is the core input of the coupling coordination model. The specific processing and application process is as follows: 1. Based on the administrative boundary vector data of 192 counties in the Yangtze River Delta urban agglomeration, the 500m resolution population urbanization and land urbanization grid data output by the U-Net model is zoned and statistics, and the average value of the urbanization grid value in each county unit is calculated. 2. The average value of the county is normalized (value range from 0 to 1), and the standardized county population urbanization index (U1) and land urbanization index (U2) are obtained. 3. The standardized U1 and U2 are directly substituted into the calculation formula of the coupling coordination model, and the county coupling degree (C) and coupling coordination degree (D) are calculated in order, which provides a quantitative basis for the subsequent coupling coordination relationship evaluation and geographical detector mechanism analysis.

#### 2.3.3. *Coupling Coordination Analysis.*

Originating from physics to characterize interaction mechanisms between systems, the coupling coordination model has been extensively applied in comprehensive assessments of regional economy, society, and resource environments [72]. This study employs this model to quantify the interaction intensity and coordination level between population urbanization and land urbanization across counties in the Yangtze River Delta urban agglomeration. By quantitatively evaluating their mutually reinforcing or constraining relationships, the approach helps reveal the matching degree and harmonious development of the population-land system, thereby providing scientific basis for understanding regional urbanization quality. Compared with conventional correlation analysis or regression models, the coupling coordination model not only captures the correlation strength between population and land urbanization, but also reflects whether their systemic development maintains a positive interaction, demonstrating distinct advantages in dynamic and comprehensive analysis [73].

The coupling coordination model is calculated as follows:


$$\begin{array}{c} C=\frac{\text{2}\sqrt{U_{\text{1}}\times U_{\text{2}}}}{U_{\text{1}}+U_{\text{2}}} \end{array}$$

$$\begin{array}{c} D=\sqrt{C\times T} \end{array}$$

In this formulation, C denotes the coupling degree, representing the intensity of interactions between the population urbanization $U_{\text{1}}$ and land urbanization $U_{\text{2}}$ subsystems. T represents the comprehensive evaluation index, typically expressed as $T=\alpha U_{\text{1}}+\beta U_{\text{2}}$, where α and β are weight coefficients generally set as α=β=0.5—indicating equal importance of both subsystems (The core goal of the coupling coordination model is to analyze the coordination relationship of human urbanization, not a single-dimensional contribution analysis. The weight setting is a classic and neutral parameter selection for coordination relationship analysis, which can avoid the impact of subjective weight bias on coordination sorting and pattern analysis). D signifies the coupling coordination degree, which comprehensively reflects both the coupling relationship between subsystems and their overall development level. Generally, a value of C closer to 1 indicates stronger interaction between the two subsystems, while a higher value of D reflects a more advanced level of coordinated development between them.

#### 2.3.4. *Geographical Detector (Geo-detector).*

Geographical detector is a statistical method designed to identify spatial heterogeneity patterns and their underlying driving factors. This technique effectively quantifies the explanatory power of individual factors on observed spatial phenomena while detecting interactive effects between multiple drivers [74]. In this study, we employ geographical detector to analyze the spatial differentiation characteristics and driving mechanisms of the population-land urbanization coupling coordination degree across counties in the Yangtze River Delta urban agglomeration. The method helps quantify the relative influence of socioeconomic, resource-environmental, and location-transportation factors on the observed coordination levels. Unlike conventional regression or correlation analysis, geographical detector operates without linear assumptions and directly utilizes variance decomposition of spatial data to assess each factor’s contribution to observed spatial heterogeneity. Additionally, it can reveal non-linear enhancement or weakening effects resulting from factor interactions [75].

The formula of geographical detector is:


$$\begin{array}{c} q=\text{1}-\frac{\sum_{h=\text{1}}^{L}N_{h}\sigma_{h}^{\text{2}}}{N\sigma^{\text{2}}} \end{array}$$

In this formula, q represents the explanatory power index, which ranges from 0 to 1. A higher q value indicates greater explanatory power of the factor regarding spatial differentiation. The variable h denotes factor stratification or category, while L represents the total number of strata. $N_{h}$ and N correspond to the sample sizes of stratum h and the total population, respectively. The terms $\sigma_{h}^{\text{2}}$ and $\sigma^{\text{2}}$ refer to the variances of stratum h and the total population, respectively.

## 3. Results

### 3.1. Land urbanization and population urbanization results

**Fig 6:**
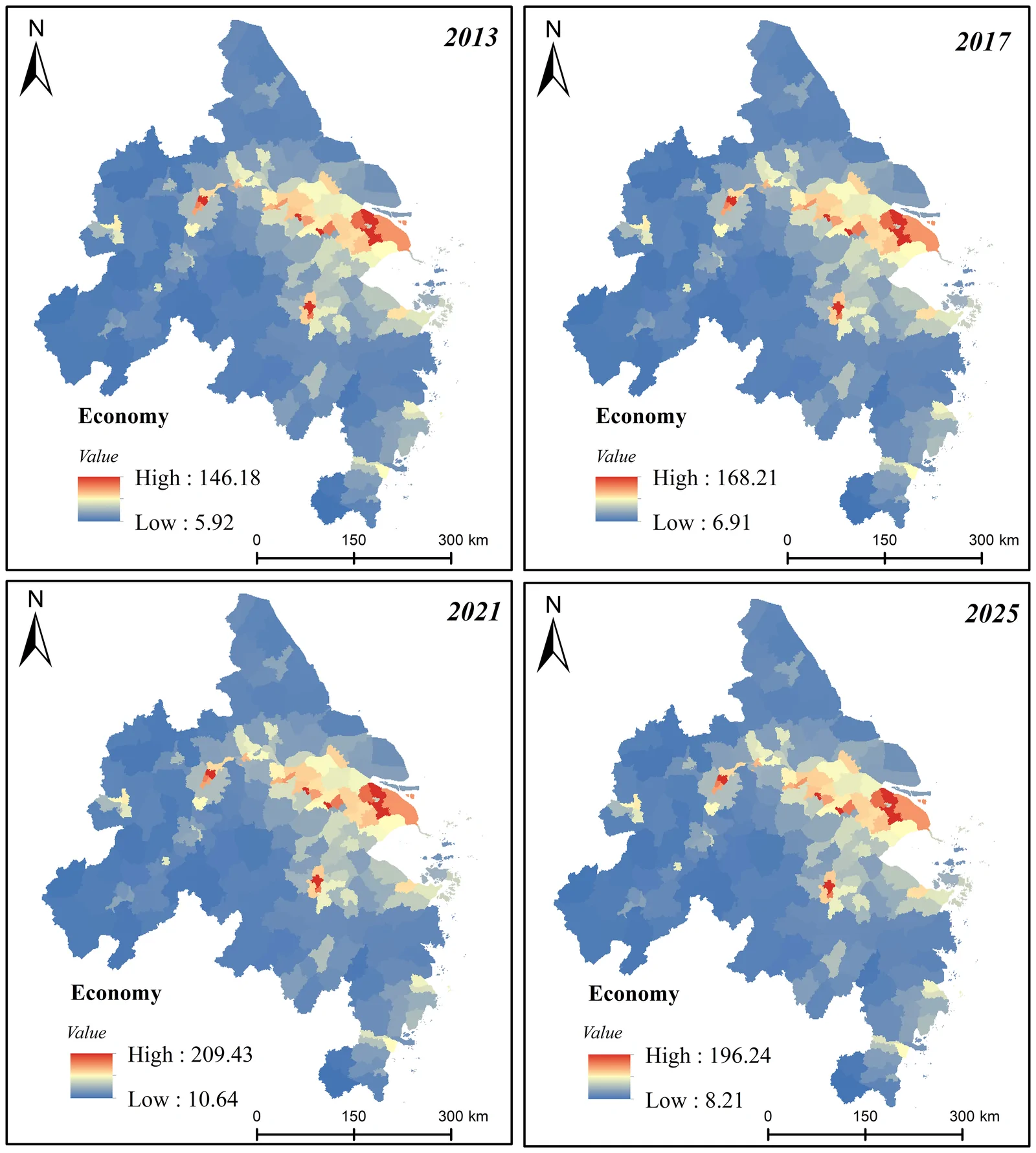
Land Urbanization Results across Counties in the Yangtze River Delta Urban Agglomeration from 2013 to 2025 (The legend shows the speed of land urbanization. Red indicates the faster, and blue indicates the slower). (Standard Map Service System of the Ministry of Natural Resources of China – Standard Map of China – Approval No. GS (2022) 1873).

**Fig 7:**
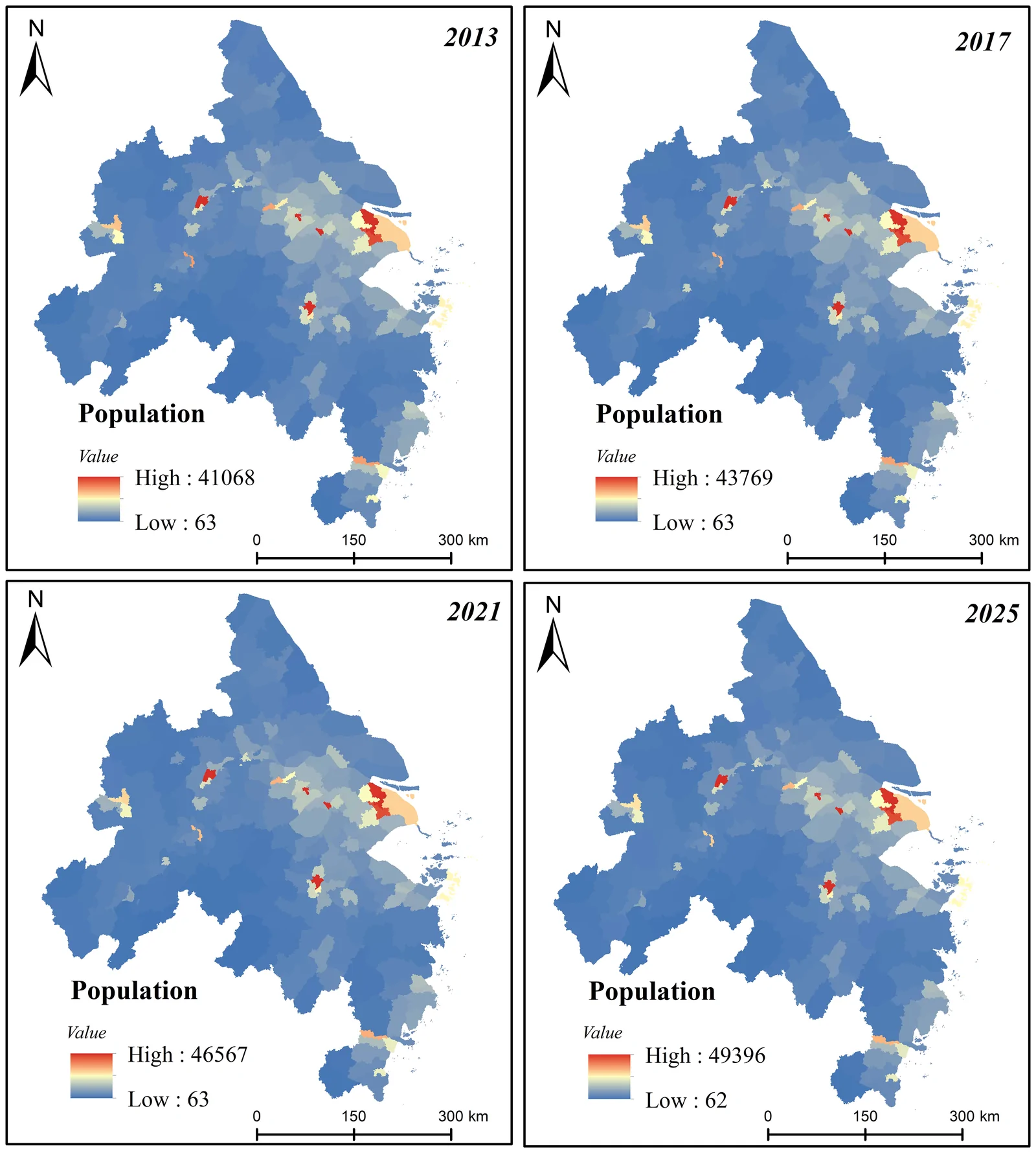
Population Urbanization Results across Counties in the Yangtze River Delta Urban Agglomeration from 2013 to 2025 (The legend shows the speed of population urbanization. Red indicates the faster, and blue indicates the slower.). (Standard Map Service System of the Ministry of Natural Resources of China – Standard Map of China – Approval No. GS (2022) 1873).

Fig 6 presents the land urbanization patterns across the Yangtze River Delta counties, derived from U-net analysis of integrated NTL and CLCD data. After the statistics and standardization of county subdivisions, the land urbanization index (U2) is obtained. Through the analysis of the integrated LandScan data and POI data through U-net, the results of the population urbanization of the county in Yangtze River Delta urban agglomeration are obtained as shown in Fig 7. After the same processing, the population urbanization index (U1) is obtained, both of which are used as the core input data for the coupling and coordination index calculation. From the perspective of overall evolution trends, the land urbanization index of counties in the Yangtze River Delta from 2013 to 2025 shows a continuous upward trend, with high-value areas gradually spreading from Shanghai, Suzhou, Wuxi, and Changzhou to the Nanjing-Hangzhou metropolitan area and the Hefei metropolitan area. The population urbanization index also maintains growth, with its spatial agglomeration characteristics being more significant, and Shanghai and its surrounding counties consistently serving as the core high-value areas for population urbanization. From the perspective of quantitative statistical characteristics, from 2013 to 2025, the mean value of the land urbanization index of counties in the Yangtze River Delta increases from 0.236 to 0.589, with an average annual growth rate of 8.25%. The variance expands from 0.032 to 0.068, and the spatial differentiation amplitude increases by 112.5%. This indicates that while land urbanization develops overall, the development gap among counties shows a continuous widening trend. The mean value of the population urbanization index increases from 0.315 to 0.627, with an average annual growth rate of 7.18%, a growth rate slightly lower than that of land urbanization. The variance expands from 0.045 to 0.072, and the spatial differentiation amplitude increases by 60.0%. The slower expansion speed of the spatial gap compared to land urbanization reflects that the spatial agglomeration characteristics of population urbanization are more stable, and the development among counties is more balanced.

Fig 7 displays the population urbanization patterns across Yangtze River Delta counties, obtained through U-net analysis of integrated LandScan and POI data. From an overall trend perspective, from 2013 to 2025, the population-land urbanization coupling coordination degree in the Yangtze River Delta exhibits an evolutionary trend of continuous increase, followed by a slight decline, and then a steady rebound. The mean value of the coupling coordination degree increases from 0.321 in 2013 to 0.635 in 2025. From 2013 to 2019, the average annual growth rate reaches 10.82%. From 2020 to 2021, influenced by adjustments in the regional development pace, the mean value slightly declines to 0.489, a decrease of 5.67% compared to 2019. From 2022 to 2025, it returns to a steady growth channel, with an average annual growth rate of 6.95%. From the perspective of county-level hierarchical structure, the number of counties with low coordination decreases from 68 in 2013–7 in 2025, with the proportion dropping from 35.42% to 3.65%. The number of counties with high and excellent coordination increases from 23 in 2013–96 in 2025, with the proportion rising from 11.98% to 50.00%. The spatial pattern of coupling coordination transitions from high values in the core and low values in the periphery to coordinated development across the entire region.

From the perspective of spatial differentiation characteristics, the variance of the coupling coordination degree decreases from 0.028 in 2013 to 0.021 in 2025, with the spatial differentiation amplitude declining by 25.0%. This indicates that the gap in coupling coordination development among counties shows a continuous narrowing trend, and the characteristic of coordinated development across the entire region gradually becomes prominent. In 2021, the variance slightly increases to 0.030, which is the maximum value during the study period, reflecting an expanded difference in the pace of coordinated development among counties during this stage. This corresponds to the feature of a decline in the mean value. After 2022, the variance continuously decreases, and the equilibrium of county development gradually recovers.

### 3.2. Coupling coordination relationship between land and population urbanization

**Fig 8:**
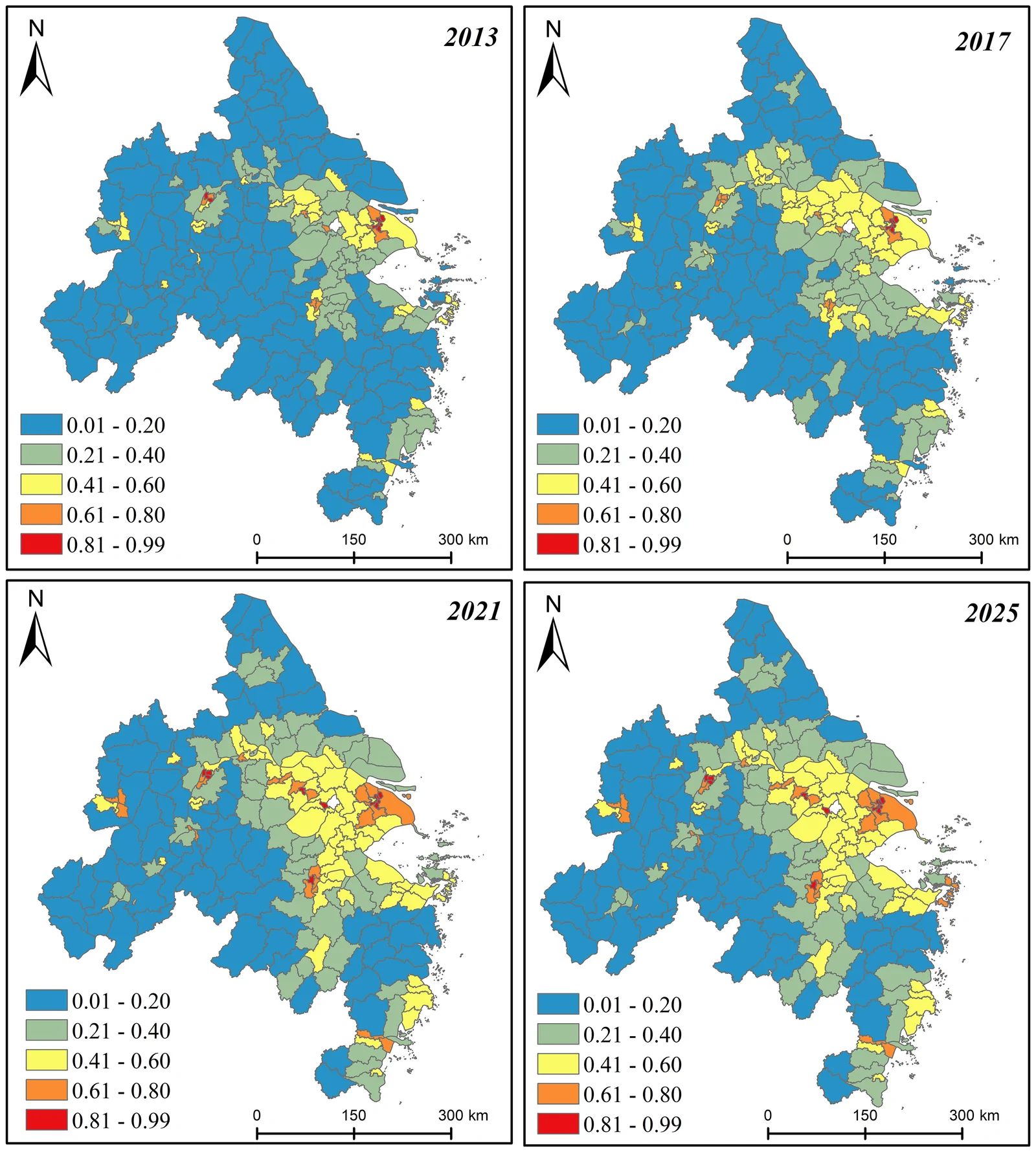
Coupling Coordination Results of Land Urbanization and Population Urbanization. (Standard Map Service System of the Ministry of Natural Resources of China – Standard Map of China – Approval No. GS (2022) 1873).

To better examine the relationship between land and population urbanization, we employ the coupling coordination degree for further analysis, with results presented in Fig 8. Temporally, the coupling coordination degree between population and land urbanization in the Yangtze River Delta counties shows continuous improvement from 2013 to 2025, indicating progressively stronger interaction and enhanced coordinated development between the two subsystems. This trend reflects substantial progress in balancing population agglomeration and land development within the urban agglomeration, driven by economic transformation and regional integration. Spatially, in 2013, high-value areas were primarily concentrated in Shanghai and counties containing core cities such as Nanjing, Hangzhou, and Ningbo, exhibiting typical point-like clustering. As regional connectivity strengthened, these high-value zones expanded into surrounding counties by 2017, forming a distinct core–periphery progression pattern. By 2021 and 2025, the high-value areas further diffused, with the eastern sector of the urban agglomeration generally achieving higher coupling coordination levels, demonstrating an areal clustering characteristic. This spatial evolution from points to areas highlights the radiating influence of core cities in fostering coordinated population–land urbanization development.

In summary, the coupling coordination between population and land urbanization in the Yangtze River Delta urban agglomeration demonstrates consistent temporal improvement and progressive spatial diffusion from core to peripheral areas. Core cities and their adjacent regions initially achieve high coordination levels, subsequently driving development in surrounding counties through economic linkages and factor mobility, ultimately forming an integrated coordinated development pattern in the eastern sector. However, the western portion and certain marginal areas of the urban agglomeration maintain relatively low coupling coordination degrees, revealing persistent spatial disparities within the region.

To quantitatively verify the judgment that population urbanization lags behind land urbanization, this study constructs a population-land urbanization ratio. If the ratio is < 1, it indicates that population urbanization lags behind land urbanization. If the ratio equals 1, it indicates that population and land urbanization are basically synchronized. If the ratio is > 1, it indicates that population urbanization is faster than land urbanization. Statistical results show that from 2013 to 2025, the mean R value of counties in the Yangtze River Delta is generally less than 1, with a multi-year average ranging from 0.89 to 0.96. This indicates that throughout the entire study period, the population urbanization index is generally lower than the land urbanization index, and the pattern of population urbanization lagging behind land urbanization at the county level stably exists. From the perspective of temporal changes, R gradually increases from 0.90 in 2013 to 1.02 in 2025. Although it gradually approaches a synchronized state, the vast majority of counties remain in the interval of R < 1 in most years. This further illustrates that the lag of population urbanization is a long-term dominant characteristic, and only a few core cities and counties gradually achieve population-land synchronization.

### 3.3. Driving mechanisms of land-population urbanization coupling coordination

Understanding the mechanisms behind the incoordination between population and land urbanization in the Yangtze River Delta counties requires a multidimensional analysis encompassing economic, social, spatial, and natural factors. Based on existing research and the actual situation of the Yangtze River Delta region, and grounded in the theory of population-land relationship territorial systems and the theory of regional unbalanced development, this study combines relevant research results on the existing population-land urbanization coupling coordination. From the four dimensions of natural foundation, economic development, social development, and spatial development, it preliminarily selects 12 candidate driving factors and constructs a candidate factor pool: Natural foundation dimension (3 factors): terrain relief, average annual precipitation, and per capita cultivated land area; Economic development dimension (4 factors): per capita GDP, proportion of secondary and tertiary industries, fixed asset investment intensity, and per capita fiscal revenue; Social development dimension (3 factors): urbanization rate of the permanent population, number of primary schools per 10,000 people, and number of medical institution beds per 10,000 people; Spatial development dimension (2 factors): construction land development intensity and traffic network density. All candidate factors ensure the continuity and availability of county-scale data during the study period (from 2013 to 2025). Based on the preliminary screening, two rounds of fine screening are conducted on the 12 candidate factors to eliminate factors with weak correlation or multicollinearity. Finally, 7 core driving factors are determined, including: economic development level, government expenditure, population agglomeration, foreign investment, urban sprawl, industrial structure, and topography. These variables collectively capture the core dynamics and constraints of county urbanization while helping to elucidate the underlying causes of asynchronous development between population and land urbanization.

County Economic Development Level serves as the fundamental driver of both population and land urbanization. Economic prosperity attracts population agglomeration to urban areas and stimulates land development demand. However, imbalanced regional development may lead to premature land expansion without corresponding population growth, creating a mismatch between land supply and demographic demand.

Government Expenditure plays a crucial role in infrastructure development and public service provision. Substantial fiscal investment enhances a county’s carrying capacity and attractiveness, facilitating coordinated population-land development. Nevertheless, when expenditure prioritizes physical infrastructure over social services, it may create disparities between land development and population needs.

Population Agglomeration directly reflects the intensity of population urbanization. The actual concentration of population not only influences land use efficiency but also determines the human-oriented quality of urban development. When population agglomeration remains insufficient, even extensive land development fails to achieve high-level coordinated development.

Foreign Investment serves as a key indicator of regional openness and industrial connectivity, significantly impacting both population and land urbanization. Substantial foreign capital typically drives industrial upgrading and creates employment opportunities, thereby attracting population inflows and stimulating land demand. However, its spatially uneven distribution may intensify regional disparities in population-land coordination.

Urban Sprawl reflects patterns of land development and spatial efficiency. When urban expansion occurs too rapidly, it often leads to extensive construction land growth without corresponding population absorption, resulting in inefficient pancake-style land urbanization that hinders coordinated population-land development.

Industrial Structure significantly influences both employment patterns and land use. Regions with a larger tertiary sector tend to attract greater population concentration, with land development prioritizing service and residential functions. In contrast, areas dominated by secondary industry often experience high land use intensity without proportional population attraction, creating a developmental imbalance.

Topography and landform: As an important constraint of natural conditions, it directly affects the potential of land for development and the spatial pattern of population agglomeration. Plain areas are more likely to form a high level of population-land coordination, while hilly or mountainous areas, due to development limitations, may experience an imbalanced state between land and population development.

Data applicability verification: This study standardizes all driving factor data (range standardization) and tests for multicollinearity through the Variance Inflation Factor (VIF). The results show that all factors have a VIF of less than 5, indicating no serious multicollinearity issues. Simultaneously, this study verifies the spatial correlation between driving factors and the coupling coordination degree. The Global Moran’s I values are all positive with P < 0.01, indicating that the data is suitable for analysis using geographic detectors. The empirical results based on the geographic detector model show that the county economic development level, government expenditure, foreign investment, and industrial structure have significant explanatory power for the population-land urbanization coupling coordination degree in the Yangtze River Delta urban agglomeration. These factors represent the core driving elements influencing the coordination of the regional population-land relationship.

Specially, in 2013, foreign investment demonstrated the strongest explanatory power, coinciding with the region’s rapid open-economy development phase. By 2017, economic development level emerged as the dominant factor. population-land. The year 2021 saw government expenditure become most influential. In 2025, industrial structure transitioned into the primary driver.

**Fig 9:**
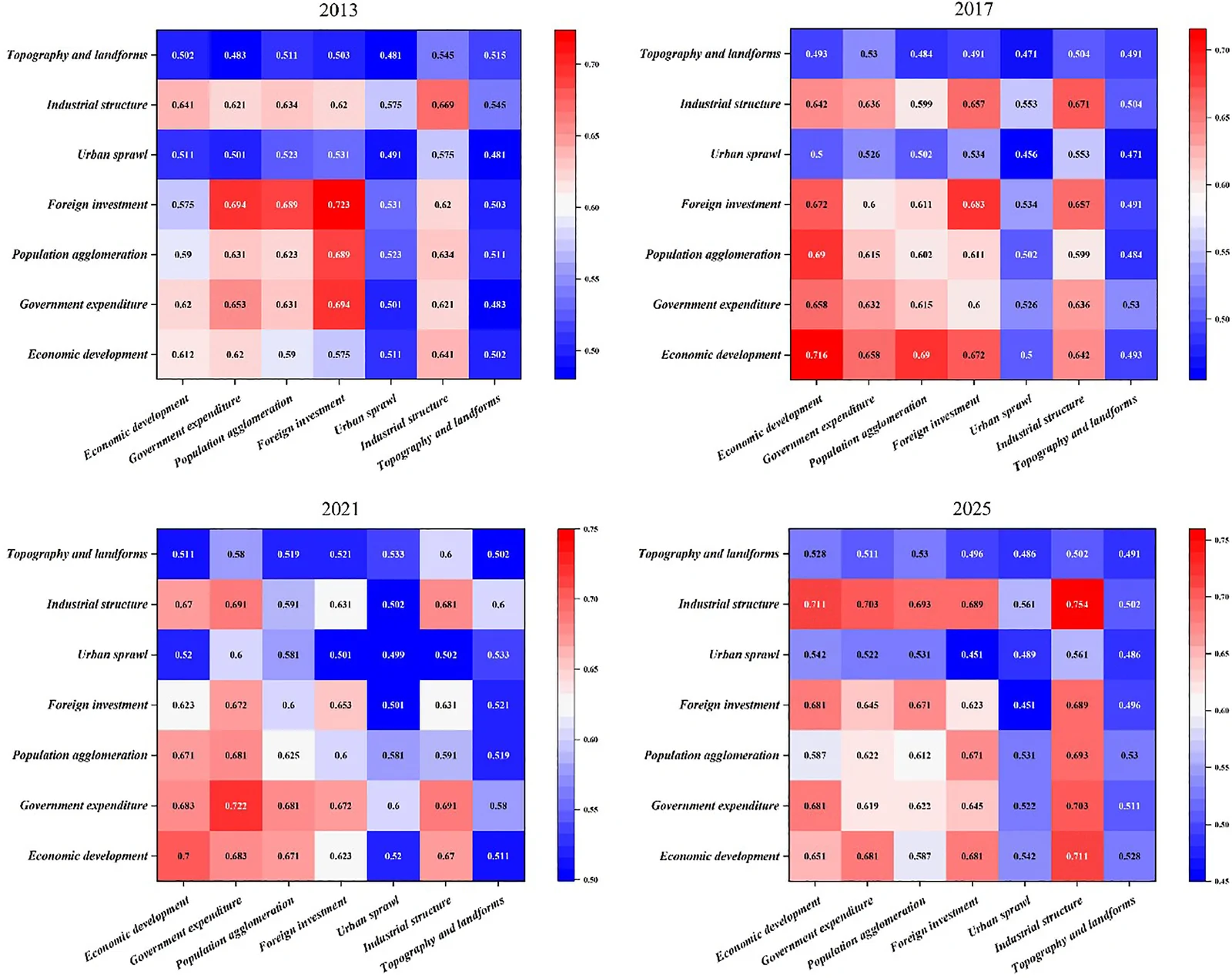
Geographical Detector Results.

The shifting dominance of driving factors across different periods reveals the dynamic evolution of population-land urbanization coordination in the Yangtze River Delta urban agglomeration. The developmental trajectory progresses from initial foreign investment-driven growth, through phases dominated by endogenous economic expansion and government regulation, before ultimately transitioning to an industrial upgrading-oriented high-quality development stage. This evolutionary pattern not only demonstrates the phased characteristics of regional urbanization coordination but also provides valuable insights for formulating differentiated policies and enhancing population-land coordination at the county level, thereby addressing potential imbalances between land and population development (Fig 9).

## 4. Discussion

Our analysis of population urbanization, land urbanization, and their coupling coordination in the Yangtze River Delta urban agglomeration counties from 2013 to 2025 reveals three key findings: land urbanization progresses significantly faster than population urbanization, the coupling coordination between them demonstrates continuous improvement, yet substantial spatial disparities persist across different periods.

Studies on population urbanization typically utilize census data and geographic big data, emphasizing spatial distribution patterns, migration dynamics, and socioeconomic impacts [76]. Western scholars frequently analyze population density configurations, employment structures, and commuting networks to explain agglomeration mechanisms, highlighting the interdependence between population urbanization and urban functional transformation [77,78]. Another research stream focuses on measuring urbanization rates, tracking spatial redistribution, and modeling population flows, with recent works incorporating emerging data sources like mobile signaling and nighttime lights to map human activity spaces [79,80]. While similarly examining population distribution, our study specifically addresses aggregation disparities at the county level, thereby refining the understanding of population urbanization processes in county contexts. Land urbanization research commonly employs multi-temporal remote sensing imagery and land cover datasets, concentrating on urban expansion rates, spatial patterns, and ecological consequences—particularly the tension between urban sprawl and sustainable development [81,82]. Chinese studies increasingly quantify construction land expansion and urban spatial structure, with recent attention to land use efficiency and functional configurations [83]. Though similarly applying multi-source data to characterize land urbanization trajectories, our work distinctively emphasizes its coordination with population agglomeration patterns.

Early studies on population-land urbanization relationships centered on the fundamental framework connecting population density, land use efficiency, and urban form. One research strand emphasizes how population agglomeration enhances land use efficiency, demonstrating that compact development and polycentric structures help mitigate urban sprawl and optimize resource allocation [84,85]. Another line investigates how land expansion influences population distribution and social equity, revealing that excessive land development without proportional population absorption leads to spatial hollowization and inefficient low-density suburban expansion [21,86]. Contemporary research increasingly contextualizes this relationship within China’s rapid urbanization experience. Studies document asynchronous development patterns between population and land urbanization—such as land preceding population or population lagging behind land—consistently showing land development outpacing population concentration [87,88]. Parallel research examines population-land coordination through coupling degree models at regional and provincial scales, incorporating institutional, policy, and economic factors into analytical frameworks [89,90]. Geographically, our study analyzes at the county scale to reveal finer-grained differentiations in population-land relationships within regions [91]. Methodologically, we combine coupling coordination modeling with geographical detector analysis to track dynamic evolution and identify driving mechanisms across temporal phases. Empirically, we find the Yangtze River Delta exhibits overall improved coupling coordination yet maintains significant spatial gradients, with foreign investment, economic development, government expenditure, and industrial structure sequentially dominating different development stages. These findings both validate existing domestic research on population-land asynchrony and elucidate its underlying dynamic mechanisms [92], while providing a Chinese empirical case that enriches international discussions on compact development and efficiency enhancement.

This study demonstrates two primary innovations. First, by integrating multi-source data to analyze outcomes of both population and land urbanization, it moves beyond conventional reliance on singular metrics like urbanization rates, thereby revealing spatial patterns and evolutionary characteristics of county urbanization with greater clarity. Second, upon identifying the mismatch between population and land urbanization, it systematically examines their coupling coordination across different periods and identifies the stage-specific influences of economic development, government expenditure, foreign investment, and industrial structure. Together, these approaches provide empirical support for spatial governance and policy optimization at the county level.

The main academic and practical contributions of this study are reflected in four aspects. First, the theoretical contribution. It integrates the three major theories of population-land relationship territorial systems, regional unbalanced development, and urban-rural integration into the study of county-level population-land urbanization coupling coordination. It constructs a theoretical analysis framework for population-land coupling coordination at the county scale and enriches the research connotation of urbanization theory at the county scale. Second, the methodological contribution. It constructs a refined measurement method for county urbanization through multi-source remote sensing data fusion. It forms an analytical system that integrates pattern, relationship, and mechanism. This provides a referable methodological reference for urbanization research at the county scale. Third, the empirical contribution. It meticulously reveals the spatiotemporal pattern and coupling coordination characteristics of population-land urbanization in counties of the Yangtze River Delta urban agglomeration from 2013 to 2025. For the first time, it identifies the dynamic evolution logic of foreign investment drive-economic endogenous growth – government regulation – industrial upgrading for population-land coordinated development in Yangtze River Delta counties. This fills a research gap in empirical studies on population-land coupling coordination at the county scale in the Yangtze River Delta. Fourth, the practical contribution. It proposes differentiated governance countermeasures targeting the spatial differences and stage characteristics of population-land coupling coordination in Yangtze River Delta counties. This provides empirical support for improving county-level spatial governance and promoting the coordinated development of population-land urbanization in the Yangtze River Delta urban agglomeration and similar regions nationwide, serving the implementation of the new-type urbanization strategy.

Based on our findings, we propose three recommendations for enhancing human-land urbanization coordination in the Yangtze River Delta counties. First, for high-coordination core areas such as Shanghai, Suzhou-Wuxi-Changzhou, and Nanjing-Hangzhou: Strictly control land expansion and promote high-quality population agglomeration. Second, for medium-coordination transition counties such as Hefei, Northern Jiangsu, and Central Zhejiang: Simultaneously promote population agglomeration and efficient land use. Third, for low-coordination lagging counties such as Southwestern Zhejiang and Northern Anhui: Prioritize making up for development shortcomings and strengthen basic guarantees.

Although this study adopts multi-source data fusion, the indicators for population urbanization and land urbanization still have measurement limitations. Furthermore, while the coupling coordination model and geographic detector are suitable for revealing the spatial-temporal patterns and the correlation strength of influencing factors, they themselves cannot robustly prove causal pathways or dynamic feedback. Simultaneously, this study still has objective limitations in the selection of proxy indicators, the construction of deep learning models, and the application and generalization of the models. These limitations are specifically reflected in three aspects: the first is the representation bias of proxy indicators, the uncertainty of deep learning labels, and the limited transferability of the models. This study uses VIIRS NTL data to represent the regional economic development level and POI density data to represent human production and living activities. Although these represent mainstream proxy indicator choices in urbanization research, inherent representation biases still exist. NTL data is easily influenced by regional industrial structures and lighting policies. Some high-energy-consumption industrial counties exhibit relatively high light intensity, making it difficult to perfectly and accurately match the actual quality of economic development. POI data is affected by the collection preferences of commercial platforms, tending to favor urban core areas and commercial facilities. Its coverage of public services and living facilities in county rural areas is insufficient, so it cannot fully reflect the characteristics of human behavior across the entire county region. Moreover, both types of indicators struggle to depict the social attributes of population and land urbanization. Second, the uncertainty of deep learning labels. When constructing the U-Net model, this study uses the fused multi-source data as input and takes the spatial distribution of land urbanization and population urbanization as supervision labels. Although this study calibrates the labels through manual visual interpretation and county-level statistical data, the labels still have a certain degree of objective uncertainty. Third, the limited transferability of the models. The wavelet transform data fusion model, the U-Net deep learning urbanization extraction model, and the mechanism analysis model incorporating coupling coordination and geographic detectors constructed in this study are all built based on the geographical characteristics, economic development levels, and urbanization stages of counties in the Yangtze River Delta urban agglomeration. The model parameters all fit the regional characteristics of the Yangtze River Delta. Therefore, the direct transferability of the models has limitations.

## 5. Conclusion

The asynchrony between population and land urbanization has become a prominent issue restricting regional high-quality development. The Yangtze River Delta, as one of the urban agglomerations with the highest urbanization level and the most developed economy in China, has a population-land coordination status at the county level that holds significant importance for the overall sustainable development of the region. Based on this, this study adopts a county-level perspective, integrates multi-source big data to construct an indicator system for population urbanization and land urbanization, uses a coupling coordination model to evaluate the matching degree between the two, and employs geographic detectors to identify key driving factors in different periods. The study finds that land and population urbanization in counties of the Yangtze River Delta urban agglomeration highly concentrates in core cities and their surrounding areas. Land urbanization expansion outpaces population agglomeration and causes a certain degree of population-land mismatch. Population and land urbanization overall show a trend of gradual coordination, but significant spatial differences and stage characteristics exist. Meanwhile, the economic development level, government expenditure, foreign investment, and industrial structure have significant explanatory power for the coupling coordination degree and exhibit differentiated dominant roles in different periods. Overall, county urbanization in the Yangtze River Delta exhibits an evolutionary characteristic of foreign investment drive government regulation industrial upgrading. This indicates that county-level population-land coordination depends not only on the overall development level but is also jointly shaped by the development stage and policy orientation. However, we can only regard this as a hypothetical evolutionary trend for a specific regional development stage, rather than a fixed and universally applicable development law.

The Yangtze River Delta has unique economic, institutional, and spatial characteristics. The conclusions of this study provide a reference for similar research on other analogous developed urban agglomerations. Overall, this study enriches the empirical understanding of the population-land coordination relationship at the county scale, improves the analytical perspective of related research, and also offers useful references and practical insights for future spatial governance and regional coordinated development at the county level in the Yangtze River Delta.
